# Two four-marker haplotypes on 7q36.1 region indicate that the potassium channel gene *HERG1 *(*KCNH2*, *Kv11.1*) is related to schizophrenia: a case control study

**DOI:** 10.1186/1744-9081-6-27

**Published:** 2010-05-28

**Authors:** Fatmahan Atalar, Tufan Tevfik Acuner, Naci Cine, Fatih Oncu, Dogan Yesilbursa, Ugur Ozbek, Solmaz Turkcan

**Affiliations:** 1 Endocrinology Laboratory, Department of Growth, Development and Pediatric Endocrinology, Child Health Institute, Istanbul University, Istanbul, Turkey; 2 Department of Genetics, Institute of Health Sciences, Istanbul University, Istanbul, Turkey; 3 Department of Medical Genetics, Faculty of Medicine, Kocaeli University, Kocaeli, Turkey; 4 Psychiatry Clinics, Turkish Ministry of Health Bakirkoy Research and Training Hospital for Psychiatry, Neurology and Neurosurgery, Istanbul, Turkey; 5 Department of Genetics, Institute of Experimental Medicine, Istanbul University, Istanbul, Turkey

## Abstract

**Background:**

The pathobiology of schizophrenia is still unclear. Its current treatment mainly depends on antipsychotic drugs. A leading adverse effect of these medications is the acquired long QT syndrome, which results from the blockade of cardiac HERG1 channels (human ether-a-go-go-related gene potassium channels 1) by antipsychotic agents. The HERG1 channel is encoded by *HERG1 *(*KCNH2*, *Kv11.1*) gene and is most highly expressed in heart and brain. Genetic variations in *HERG1 *predispose to acquired long QT syndrome. We hypothesized that the blockade of HERG1 channels by antipsychotics might also be significant for their therapeutic mode of action, indicating a novel mechanism in the pathogenesis of schizophrenia.

**Methods:**

We genotyped four single nucleotide polymorphisms (SNPs) in 7q36.1 region (two SNPs, rs1805123 and rs3800779, located on *HERG1*, and two SNPs, rs885684 and rs956642, at the 3'-downstream intergenic region) and then performed single SNP and haplotype association analyses in 84 patients with schizophrenia and 74 healthy controls after the exclusion of individuals having prolonged or shortened QT interval on electrocardiogram.

**Results:**

Our analyses revealed that both genotype and allele frequencies of rs3800779 (c.307+585G>T) were significantly different between populations (*P *= 0.023 and *P *= 0.018, respectively). We also identified that two previously undescribed four-marker haplotypes which are nearly allelic opposite of each other and located in chr7:150225599-150302147bp position encompassing *HERG1 *were either overrepresented (A-A-A-T, the at-risk haplotype, *P *= 0.0007) or underrepresented (C-A-C-G, the protective haplotype, *P *= 0.005) in patients compared to controls.

**Conclusions:**

Our results indicate that the potassium channel gene *HERG1 *is related to schizophrenia. Our findings may also implicate the whole family of HERG channels (HERG1, HERG2 and HERG3) in the pathogenesis of psychosis and its treatment.

## Background

Schizophrenia (SCH) is a serious mental disorder affecting around 0.5% of the general population worldwide [1]. Despite being a clinically recognized entity for more than a century, the etiopathogenesis of SCH still remains a mistery [1,2]. SCH seems to be a multifactorial disorder, in which the contribution of both genetic and environmental factors and their interplay are important [1,3]. Little is known about the underlying environmental factors, and the rare genetic variants so far disclosed as susceptibility factors are neither necessary nor sufficient for the disease [1]. As yet, there is no identified biological marker with which a biologically valid diagnosis can be made [1,2]. The current treatment depends on antipsychotic drugs which have antidopaminergic properties (D2 receptor blockade) as the main feature, with or without a certain degree of additional antiserotonergic effects (5-HT2A receptor blockade) in particular [4,5]. The high remission but low recovery rate by antipsychotic therapy [6,7] is probably due to their insufficient specificity for the complex mechanism underlying SCH, which might go beyond the most frequently-described hyperdopaminergic pathophysiology [2,8]. The unspecificity in dopaminergic-blocking effects of antipsychotic drugs also seems to be the cause of their main side-effects, the extrapyramidal syndromes (EPS) [9,10]. A further leading drug-induced adverse effect is the acquired long QT (LQT) syndrome, which results from the blockade of a type of voltage-gated potassium channel in the heart, namely the cardiac HERG1 (human ether-a-go-go-related gene potassium channel 1) channel, by antipsychotic agents [11-13].

**Figure 1:**
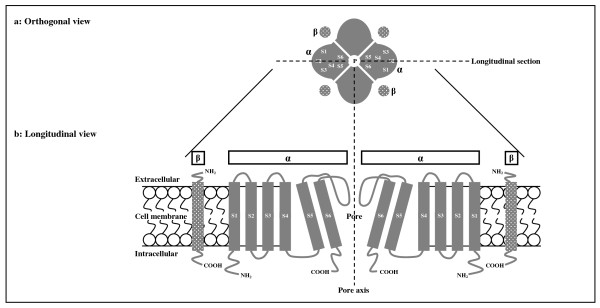
**Tetrameric organization and membrane topology of HERG1 channel**. **a: **Orthogonal view from the extracellular side demonstrating the tetrameric organization of HERG1 channel. **b: **Longitudinal view through the channel, taken from the longitudinal section shown at the top, displaying the membrane topology of only two reciprocal components across the pore of the tetrameric HERG1 channel. The channel exhibits a tetrameric composition of one main alpha-subunit (HERG1 protein, shown as α and in plain gray) and one auxiliary beta-subunit (minK or MiRP1 protein, shown as β and in dotted gray). The alpha-subunit is composed of six transmembrane-spanning domains (labelled S1-S6), S1-S4 being the voltage-sensing domain and S5-S6 the pore-forming domain. The beta-subunit comprises one transmembrane-spanning domain. The NH_2 _and COOH termini are also shown. α, alpha-subunit; β, beta-subunit; P, pore.

HERG1 (also referred as KCNH2 or Kv11.1) belongs to a particular subtype known as H or Kv11 subfamily of the voltage-gated potassium channels [14], along with HERG2 (KCNH6, Kv11.2) and HERG3 (KCNH7, Kv11.3) [15]. According to the mostly stated view in literature, the HERG channels characteristically exhibit a tetrameric composition of one main alpha-subunit (HERG1, HERG2 or HERG3 protein), which comprises six transmembrane-spanning domains denoted S1-S6, and one auxiliary beta-subunit (minK, MiRP1 or MiRP2 protein) (Figure 1) [11,16-18]. The co-assembly of HERG channels through different alpha-subunits of the same subfamily, alternative isoforms of a given alpha-subunit or diverse auxiliary beta-subunits gives rise to their enormous structural and functional variability among various tissues and cells as well as among individuals [19-21]. These channels play an important role in the repolarization of the cellular membrane potential of excitable cells, such as cardiac, neuronal and smooth muscle cells [16]. Hence, HERG1 is expressed at the highest levels in heart and brain, whereas HERG2 and HERG3 are expressed exclusively in brain [22-25]. Notably, HERG1 channel is responsible for the rapid delayed rectifier current (I_Kr_), which is a major component of the repolarization of cardiac action potentials [16,18]. The HERG1 protein, alpha-subunit of the HERG1 channel, is encoded by *HERG1 *gene, which contains 15 exons on 7q36.1 region and gives rise to at least three distinct isoforms by alternative splicing (the canonical form, 4282 bp) [26,27]. To date, more than 300 genetic variations in *HERG1 *have been found either to cause the congenital form or to predispose to the acquired form of the so-called LQT and short QT (SQT) syndromes [28-30]. These syndromes are characterized by cardiac arrhythmias and a prolonged or shorthened QT interval on electrocardiogram (ECG), which reflects the duration of cardiac repolarization [29,30]. Whilst the congenital form of the syndromes comprises inherited phenotypes caused by *HERG1 *variations, the acquired form encompasses reversible phenotypes induced most often through the blockade of HERG1 channels, with or without an underlying predisposition resulting from *HERG1 *variations, by certain drugs, in particular the antipsychotics [12,30,31].

An old and enduring notion in psychiatry is that the therapeutic and adverse effects of antipsychotic drugs frequently co-occur [32,33], indicating that they work through more or less similar or overlapping mechanisms. This is true in the case of their antidopaminergic properties which produce therapeutic effects on one hand, but cause EPS side effects on the other [10,34]. By analogy, we hypothesized that the blockade of HERG1 channels by antipsychotics, which is responsible for the acquired LQT side effects, might also be involved in the therapeutic mechanism of these drugs, thereby providing a clue to the core pathogenesis of SCH. We therefore studied a number of genetic variations in and around the *HERG1 *gene by single SNP and haplotype association analyses in a population of patients with SCH. Recently, Huffaker et al. [35] studied two family-based cohorts of European ancestry and three case-control cohorts also of European ancestry (Germans, Armenians and Italians). They identified six single nucleotide polymorphisms (SNPs) to be associated with SCH in family-based studies, four SNPs in the German case-control study and four SNPs in the meta-analysis including all five clinical data sets, and reported *HERG1 *as a previously undescribed potential susceptibility gene for SCH [35].

## Methods

### Patients and controls

The study population consisted of 87 unrelated Turkish male inpatients with SCH (mean age = 34,91 ± 6,98 years) recruited from the Bakirkoy Research and Training Hospital for Psychiatry, Neurology and Neurosurgery (BRSHH) in Istanbul, a specialized referral center of the Turkish Ministry of Health. The inclusion criteria were to be aged between 18 and 65 years, to have an informed written consent by themselves and/or by their legal supervisors and to have a diagnosis of SCH. The diagnosis of SCH was made in accordance with the Diagnostic and Statistical Manual of Mental Disorders - Fourth Edition (DSM-IV) in consensus by two psychiatrists who interviewed the patients independently using the Structured Clinical Interview for DSM-IV (SCID-I) [36] Cases were screened to exclude substance-induced psychotic disorder, psychosis due to a general medical condition, and co-morbid psychiatric disorder. The control population included 77 Turkish males (mean age = 34,23 ± 8,20 years) collected from the Control Subjects Biobank at the Research Institute for Experimental Medicine of Istanbul University. None of the controls in that biobank has a known or identified psychiatric disorder, neurological disease or medical condition. Patients and controls were recruited from Istanbul metropolitan area. Both study populations do well represent the general population of Turkey as Istanbul receives substantial internal migration from almost all regions of Turkey since 1950s.

### Exclusion of subjects with a long or short QT interval on electrocardiogram (ECG)

As many genetic variations in the *HERG1 *gene are related with congenital or acquired forms of LQT and SQT syndromes, we excluded the patients and controls that had a prolonged or shortened QT interval on their ECG recordings (the main diagnostic criterium for LQT or SQT syndromes, respectively [29]) in order to discard any incidental confounding effect which could arise from a putative *HERG1*-LQT/SQT association rather than a real *HERG1*-SCH relationship. A standard 12-lead resting ECG was obtained from each patient and control with an automated electrocardiograph (ECG-9620 Cardifax, Nihan Kohden, Japan) at a paper speed of 25 mm/s. The QT interval and the so-derived QTc value (corrected QT according to heart rate by the Bazett's formula [29,37], QTc = QT/√RR) were measured automatically by the Modular ECG Analysis System (MEANS). In the international guidelines [38,39] or related literature [13,29], the widely accepted threshold values are QTc > 450 ms in men for prolonged QT interval and QTc < 300 ms for shortened QT interval. ECG recordings of the patients and controls were evaluated accordingly, and three patients and three controls having QTc > 450 ms or < 300 ms were excluded. The remaining 84 patients (mean age = 34,37 ± 6,70 years) and 74 controls (mean age = 34,32 ± 8,11 years) formed the ultimate study groups.

### Selection of single nucleotide polymorphisms (SNPs) for association analyses

**Figure 2:**
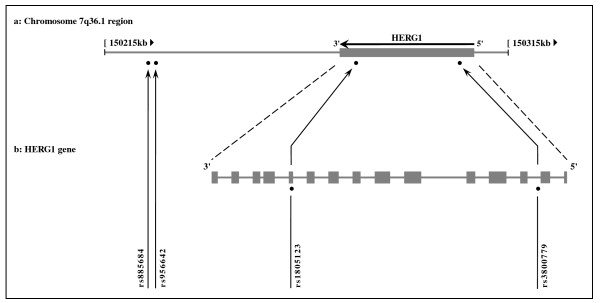
**Location of the four SNPs analyzed in and around the *HERG1 *gene on 7q36.1 region**. **a: **The 100 kb genomic region (shown as horizontal gray line) including the *HERG1 *gene (shown as gray rectangle) on 7q36.1. **b: **The organization of exons (shown as gray boxes) and introns (shown as horizontal gray lines) of the *HERG1 *gene. The four SNPs analyzed span ~76 kb on genomic DNA in chr7:150225599-150302147 bp position. Two SNPs (rs885684 and rs956642) are located at the 3'-downstream intergenic region of *HERG1*, and the other two SNPs (rs1805123 and rs3800779) are located respectively in exon 11 and intron 2 of the gene. Black circles (●) represent the SNP locations, and the rs numbers of SNPs are shown along the relevant arrows. SNP, single nucleotide polymorphism.

A large-scale, two-step design, population-based European study (KORA), conducted by Pfeufer et al. [40], was previously performed in order to determine the associations between genetic variations in four potassium channel genes, including *HERG1*, and QT interval prolongation. In the present work, we investigated four out of five SNPs, located in and around *HERG1*, that were found to be significantly associated with QT interval prolongation in the screening phase of the mentioned study. In order to minimize false-negative error rate, we chose the SNPs from the "screening" rather than the subsequent "confirmation" phase. We excluded the fifth SNP found to be significant in the study of Pfeufer et al. [40] as it was located within another gene (*NOS3*), 5'-upstream of *HERG1*. Among the four SNPs intended to be analyzed in our study, two were located on *HERG1 *gene (rs1805123 in the exon 11, and rs3800779 in the intron 2), and the other two SNPs (rs885684 and rs956642) were located at the 3'-downstream intergenic region of *HERG1 *(see Figure 2). The SNPs span ~76 kb on genomic DNA in chr7:150225599-150302147 bp position.

### DNA extraction and SNP genotyping

Genomic DNA was extracted from 10 ml fresh peripheral blood using the QIAamp DNA Blood Mini Kit (Qiagen, Germany) according to the manifacturer's protocol. Twenty microliters of genomic DNA from each patient and control subject were prepared at a concentration of 2 ng/L in v-bottomed 96-well microtitre plates. Repeat and blank samples were also included in the plates as experimental controls. The microtitre plates were then transported to the KBiosciences Company, Cambridge, UK, via a private courier and in accordance with the national and international legal regulations and technical requirements for international transfer of biological materials. All of the genotyping was performed by the KBiosciences using the KASP technology, which is a competitive allele-specific PCR incorporating a FRET quencher cassette, according to the protocol used by the company [41].

### Statistics for single SNP and haplotype association analyses

Deviations from Hardy-Weinberg Equilibrium (HWE) were assessed for quality control of genotyping procedures among patients and controls separately. SNPs were excluded from the analysis if they were out of HWE (*P *< 0.05) or had a minor allele frequency of less than 5%. The allele and genotype frequencies were obtained by direct counting. We used SPSS 11.5 software for statistical analyses. The *P *values were corrected by means of Bonferroni correction for multiple testing. We used Haploview software [42] to reconstruct haplotypes and estimate haplotype frequencies in the unrelated patients and controls. In order to obtain a measure of significance corrected for multiple testing, we ran 10000 permuations to compute *P *values using the Haploview program. Comparisons of the distributions of the allele, genotype and haplotype frequencies were performed using the chi-square test. Statistical significance was defined as *P *< 0.05.

### Ethical issues

This study was approved by the Ethics Committee of BRSHH, and was in compliance with the World Medical Association (WMA) Declaration of Helsinki. Written informed consent was obtained from all subjects and/or their legal supervisors.

## Results

### Single SNP association analysis

The χ^2 ^goodness-of-fit test showed that the four SNPs genotyped were within the distribution expected according to HWE in both the patient (rs885684: χ^2 ^= 3.728, *P *= 0.053; rs956642: χ^2 ^= 0.705, *P *= 0.401; rs1805123: χ^2 ^= 1.185, *P *= 0.276; rs3800779: χ^2 ^= 0.247, *P *= 0.619) and the control (rs885684: χ^2 ^= 3.151, *P *= 0.075; rs956642: χ^2 ^= 0.786, *P *= 0.375; rs1805123: χ^2 ^= 0.035, *P *= 0.852; rs3800779: χ^2 ^= 3.736, *P *= 0.053) populations. The genotype and allele frequencies of the individual SNPs and the results of the association analyses between patients and controls are summarized in Table 1. The minor allele frequencies were more than 5%. Neither the genotype nor the allele frequencies of rs885684, rs956642 and rs1805123 differ between patients and controls. However, both the genotype and allele frequencies of rs3800779 (c.307+585G>T) were significantly different between the populations (see Table 1). The GG genotype was found to be less frequent and GT and TT genotypes more frequent, whereas the frequency of the G allele was lower and the frequency of the T allele was higher in patients than in controls (*P *= 0.023 and *P *= 0.018, respectively).

**Table 1:** Genotype and allele frequencies and results of the association analysis between patients and controls.

SNP	Genotype frequencies, n (%)			χ^2^	*P*-value	Allele frequencies			χ^2^	*P*-value
						
	Genotype	SCH (n = 84)	CTR (n = 74)			Allele	SCH (n = 84)	CTR (n = 74)		
**rs885684**	AA	37 (44.0)	34 (45.9)	1.06	0.302	A/C	0.696/0.304	0.642/0.358	1.06	0.303
								
	AC	43 (51.2)	27 (36.5)							
								
	CC	4 (4.8)	13 (17.6)							

**rs956642**	AA	37 (44.0)	28 (37.8)	2.38	0.123	A/G	0.679/0.321	0.595/0.405	2.41	0.120
								
	AG	40 (47.6)	32 (43.2)							
								
	GG	7 (8.3)	14 (19.0)							

**rs1805123**	AA	51 (60.7)	39 (52.7)	2.12	0.145	A/C	0.792/0.208	0.723/0.277	2.03	0.153
								
	AC	31 (36.9)	29 (39.2)							
								
	CC	2 (2.4)	6 (8.1)							

**rs3800779**	GG	37 (44.1)	49 (66.2)	5.15	**0.023**	G/T	0.673/0.327	0.791/0.209	5.52	**0.018**
								
	GT	39 (46.4)	19 (25.7)							
								
	TT	8 (9.5)	6 (8.1)							

SNP, single nucleotide polymorphism; SCH, patients with schizophrenia; CTR, control subjects.

Alleles shown are major/minor.

The *P*-values given are corrected by means of Bonferroni correction.

Association reaching nominal significance (*P *< 0.05) is shown in bold.

### Haplotype association analysis

**Figure 3:**
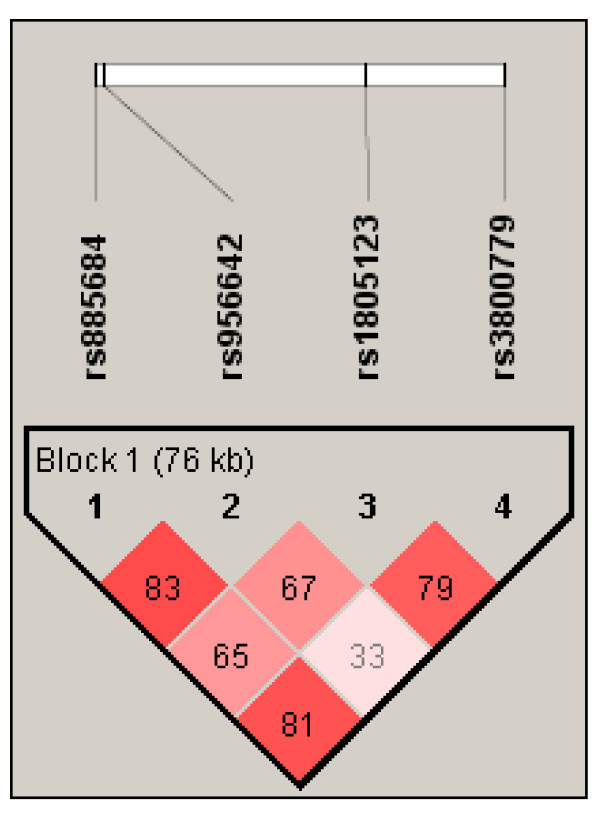
**Linkage disequilibrium pattern of the SNPs along the 7q36.1 region encompassing the *HERG1 *gene**. The graphic illustrates the one distinct haplotype block defined using the Haploview program. The linkage disequilibrium (D') between any two SNPs is shown in the cross cell. The darker the color indicates the higher linkage disequilibrium between any two SNPs. The linkage disequilibrium (*r^2^*) between any two SNPs is as follows: rs885684-rs956642: 0.191; rs885684-rs1805123: 0.277; rs885684-rs3800779: 0.122; rs956642-rs1805123: 0.081; rs956642-rs3800779: 0.072; rs1805123-rs3800779: 0.074. SNP, single nucleotide polymorphism.

Pair-wise linkage disequilibrium (LD) was calculated between all pairs of SNPs using the Haploview program and the results are shown in Figure 3. All four SNPs were part of one haplotype block and fairly tight LD was observed in most SNP pairs (see Figure 3). Analyses of sliding windows of two- and three-SNP haplotypes did not reveal any significant difference in haplotype distributions between patients and controls (data not shown). Four-SNP haplotypes with frequencies ≥1% are listed in Table 2. The analysis of the four-SNP haplotypes displayed significant differences in the distributions of two haplotypes, A-A-A-T and C-A-C-G, between the populations (see Table 2). The A-A-A-T haplotype was overrepresented, whereas the C-A-C-G haplotype was underrepresented in patients as compared to controls (*P *= 0.0007, *P *= 0.005, respectively), suggesting the former to be a high-risk and the latter a low-risk haplotype. It is of note that the potentially "protective" haplotype C-A-C-G is an allelic opposite at all loci but the second locus of the potentially "at-risk" haplotype A-A-A-T. The C-G-C-G haplotype that is the exact allelic opposite of the A-A-A-T haplotype was absent in our patient and control populations.

**Table 2:** Four-SNP haplotype frequencies and results of the association analysis between patients and controls.

Haplotype	Frequency	Case, control ratios	χ^2^	*P*-value
A-A-A-G	0.206	0.217, 0.194	0.26	0.610

C-A-C-G ^1^	0.177	0.121, 0.241	7.79	**0.005**

A-G-A-G	0.177	0.146, 0.212	2.36	0.124

A-G-A-T	0.139	0.114, 0.167	1.81	0.178

C-A-A-G	0.126	0.138, 0.112	0.49	0.484

A-A-A-T ^1^	0.094	0.147, 0.035	11.46	**0.0007**

A-A-C-T	0.020	0.033, 0.005	3.12	0.077

A-G-C-G	0.019	0.017, 0.022	0.10	0.752

A-A-C-G	0.017	0.024, 0.008	1.25	0.263

C-G-A-T	0.012	0.022, 0.002	2.52	0.112

SNP, single nucleotide polymorphism.

Case refers to patients with schizophrenia, and control refers to control subjects.

Haplotypes listed are only those with a frequency ≥1%.

The *P*-values given are based on 10000 permutations using the Haploview program.

Associations reaching nominal significance (*P *< 0.05) are shown in bold.

^1^Note that C-A-C-G haplotype is an allelic opposite at all, but the second, loci of A-A-A-T haplotype. C-G-C-G haplotype that is the exact allelic opposite of A-A-A-T haplotype was absent in our patient and control populations.

## Discussion

In this study, we reasoned that a particular mechanism (HERG1 channel blockade, for example) responsible for a prevailing adverse effect of antipsychotic drugs (acquired LQT in this case) is also likely to be one unique mechanism by which their therapeutic effects are mediated, and that, by inference, this might contribute to our understanding of the underlying mechanisms in SCH. This assumption led us to investigate the putative association of *HERG1 *gene with SCH. To discard any incidental confounding association of *HERG1 *with LQT or SQT syndromes among study and control populations, we excluded those patients and controls with LQT or SQT intervals on ECG. We chose the SNPs to be analyzed (two located on *HERG1 *and two at the 3'-downstream region of the gene) on the basis of their association with QT interval prolongation according to a large-scale, two-step design, population-based European study [40]. Our analyses revealed that two haplotypes, which were nearly allelic opposite of each other and composed of the four SNPs investigated, were either overrepresented (A-A-A-T, the at-risk haplotype) or underrepresented (C-A-C-G, the protective haplotype) in patients with SCH as compared to control subjects. Because the relevant haplotype block which spans a ~76 kb region of genomic DNA in chr7:150225599-150302147 bp position does not include any other genes, this finding might be essentially attributed to either *HERG1 *or its surrounding regulatory sequences. In support of this, we report that one of the two SNPs located on *HERG1 *(rs3800779 in the intron 2, c.307+585G>T) shows significant differences in both genotype and allele distributions between patients and controls. Our results thereby identify the *HERG1 *gene as a susceptibility factor for SCH and implicate the contribution of HERG1 channels to its pathobiology.

Despite intensive research, the pathobiology of SCH still remains obscure [1,2]. The bulk of current evidence gathered from a wide range of studies suggests that SCH is a disorder of neurodevelopment involving the dysfunction of multiple neurotransmitters in neural circuits particularly concerning the dopaminergic, serotonergic, glutamatergic and GABAergic systems within or between cortical-prefrontal and subcortical-limbic regions [2,7,43,44]. The biological characteristics of HERG channels are broadly in concordance with the above-mentioned features of SCH pathobiology. *ERG1 *(this is homologous to the human gene *HERG1*, referring its orthologs in all species) is expressed at high levels throughout the brain besides the heart, specifically, in the hippocampus, neocortex, hypothalamus, thalamus, amygdala, substantia nigra, red nucleus and cerebellum [22,23,25,45,46]. The other two members of the *ERG *genes family, *ERG3 *and *ERG2 *(homologous terms in all species for *HERG3 *and *HERG2*, respectively), also have a more or less widespread expression in the brain [22,23,45,46] and moreover they seem to be nervous system-specific [24]. There is convincing evidence that the products of these three genes form heterotetramers in various combinations at specific brain regions where they co-expressed [45-48], giving rise to multiple heterotetrameric ERG channels with functional properties distinct from those of homotetramers [21,47-49]. All types of ERG channels studied so far, and particularly the most studied ERG1 channels, were shown to modify neuronal excitability and spike frequency adaption [49-53]. *ERG1 *gene was consistently shown to be expressed in dopaminergic [54] and serotonergic [47] neurons from rat brain and in GABAergic interneurons from mouse [45] and rat brains [22,46]. It is of particular note that both an experimental study [54] and a computational model [55] showed the modulation of dopamine neurons by ERG channels. The neuronal expression of all three *ERG *genes, in particular including the most studied *ERG1*, was found to be developmentally regulated [23,56].

Recently, Huffaker et al. [35] reported *HERG1 *as a previously undescribed potential susceptibility gene for SCH. The authors genotyped, in a family-based association study, the haplotype-tagging SNPs in 10 previously reported candidate genes and their results revealed the 7q36.1 region to be strongly associated with SCH. They then performed initial studies of 43 or 40 SNPs in the 7q36.1 region of two family-based cohorts of European ancestry. Subsequently, seven SNPs in *HERG1*, selected according to initial results, were analyzed in three case-control cohorts, also of European ancestry (Germans, Armenians and Italians). They identified six SNPs to be associated with SCH in family-based studies, four SNPs in the German case-control study and four SNPs in the meta-analysis including all five clinical data sets, although none of the SNPs were significant in the Armenian and Italian samples. Interestingly, across all their samples, the *HERG1 *SNP most strongly associated with SCH was rs3800779, which is also the SNP showing unique significance towards the same direction of association in our study. Nevertheless, their three-marker haplotype analysis did not show an association that was more significant than individual SNPs. The authors also investigated *HERG1 *expression in brain and found expression of full-length isoform 1A (*KCNH2*-1A or *HERG1*-1A) to be significantly lower in patients with SCH than in controls, but they were unable to detect an association with the *HERG1 *risk genotypes that they identified. Following further investigation aimed at determining the apparently complicated gene processing, they ultimately discovered a previously undescribed brain-specific isoform (*KCNH2*-3.1 or *HERG1*-3.1) which lacks the first two exons and introns of the full-length gene but contains the downstream region. Furthermore, they determined that expression of *HERG1*-3.1 was increased in brain of patients compared to controls and that this was significantly associated with *HERG1 *risk genotypes. Taken together, the authors argued that overexpression of this newly identified isoform is related to the pathogenesis of SCH, and that the mechanism by which the disease-associated SNPs contribute to increased risk involves the regulation of *HERG1*-3.1 transcription by a splicing mechanism yet to be determined.

**Figure 4:**
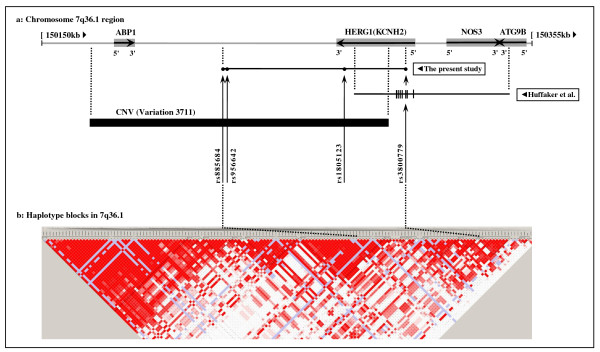
**Organization of genomic region and localization of haplotype blocks situated between positions 150150-150355 kb in 7q36.1**. **a: **A 205 kb part situated between positions 150150-150355 kb of the 7q36.1 genomic region (gray horizontal line at the top), along with the genes *ABP1*, *HERG1 *(*KCNH2*), *NOS3 *and *ATG9B *located here (gray boxes), is shown. Relative position on 7q36.1 (marked by dotted lines) of the region investigated in the present study (~76 kb of length in chr7:150225599-150302147 bp position) is represented by the upper horizontal black line and that of Huffaker et al. (~65 kb of length in chr7:150280464-150345679 bp position; see text for the reference) by the lower horizontal black line. Black circles (●) on the upper horizontal black line indicate the locations of four SNPs analyzed in the present study, and the rs numbers of SNPs are presented along the relevant arrows. Small vertical lines (∣) on the lower horizontal black line indicate the locations of seven SNPs analyzed in all cohorts studied by Huffaker et al. rs3800779 is the single common SNP investigated in both studies and showed the higher significance among six significant SNPs found in the study of Huffaker et al. and the unique significance among four SNPs analyzed in the present study. Black horizontal rectangle at the bottom shows the position on 7q36.1 (marked by dotted lines) of the copy number variation (CNV, Variation 3711) of ~125 kb of length located in this part of the genome between positions 150169754-150294630 bp and partly inserted into *HERG1 *(~21.5 kb from its 3'-end). Three out of four SNPs (except rs3800779) that composes the schizophrenia-associated haplotypes A-A-A-T/C-A-C-G identified in the present study are located within this CNV. **b: **Haplotype blocks defined between positions 150150-150355 kb in 7q36.1 genomic region in the CEU population of the International HapMap Project http://hapmap.ncbi.nlm.nih.gov are shown. As marked by dotted lines, the schizophrenia-associated A-A-A-T/C-A-C-G haplotypes identified in the present study extend across a number of defined haplotype blocks. *ABP1*, amiloride binding protein 1 gene; *NOS3*, nitric oxide synthase 3 (endothelial cell) gene; *ATG9B*, nitric oxide synthase 3 antisense gene; SNP, single nucleotide polymorphism; CNV, copy number variation.

Although our starting point was different from that of Huffaker et al. [35] (hypothesis-driven in our case and evidence-based in theirs), we identified the same gene, *HERG1*, in association with SCH. The 7q36.1 regions investigated by us (~76 kb) and by Huffaker et al. [35] (~65 kb) are presented in Figure 4a. The two regions overlap well on *HERG1*, however that investigated by Huffaker et al. [35] extends in the 5'-upstream direction, whilst ours extends towards the region 3'-downstream of the gene. As an independent research group we confirm Huffaker et al. [35] regarding the association of SCH with *HERG1 *and specifically the *HERG1 *SNP rs3800779 which shows higher significance in their study and unique significance in ours towards the same direction of association. We also present the association of *HERG1 *with SCH in Turkish population, as was shown by Huffaker et al. [35] in American and German populations, but not in Armenians and Italians. In addition to single SNP association, we furthermore report two previously undescribed SCH-associated four-marker haplotypes on 7q36.1 region encompassing *HERG1*, that were more significant than unique significant SNP (the at-risk haplotype A-A-A-T and the protective haplotype C-A-C-G). Our study differed from Huffaker et al. [35] by excluding patients and controls having LQT or SQT interval on ECG recordings, in order to discard any incidental confounding association of *HERG1 *with underlying LQT or SQT syndromes which could have remained occult within the study and control groups. This provided to us more specific and statistically significant findings, in particular the identification of two unique SCH-associated haplotypes (A-A-A-T/C-A-C-G), nearly allelic opposite of each other. This specific finding further widens the implications introduced by Huffaker et al. [35] regarding the possibilities of a more extended involvement of genes in 7q36.1 other than *HERG1 *and of a more complicated regulation of *HERG1 *expression other than splicing mechanism in SCH. As concerns our first inference, the A-A-A-T/C-A-C-G haplotypes extending across a number of haplotype blocks defined in the CEU population (Utah residents in USA with Northern and Western European ancestry from the *Centre d'Etude du Polymorphisme Humaine *collection) of the International HapMap Project [42] (Figure 4b) towards the intergenic region 3'-downstream of *HERG1 *may be a part of even larger SCH-associated haplotypes that might be related with more than one gene (Figure 4a) and/or with the copy number variation (CNV) of ~125 kb of length (Variation 3711) in 7q36.1. With regard to our second inference, the A-A-A-T/C-A-C-G haplotypes may also signify the contribution of CNV 3711, which is partly inserted into *HERG1 *(~21.5 kb from its 3'-end, Figure 4a), to the pathogenesis of SCH by affecting gene expression, which is a well-known mechanism of CNVs [57,58]. Taken together, further to the comments of Huffaker et al. [35] on their results, our findings make it plausible a more extended involvement of 7q36.1 region apart from *HERG1 *and a more complicated regulation of *HERG1 *transcription other than splicing mechanism in SCH.

Our study also justifies the usefulness of our preliminary rationale envisaging that both an adverse and a therapeutic effect of antipsychotic drugs might depend on a common mechanism, which could enhance our knowledge about the pathobiology of SCH. Although for many years it was believed that the prevalent EPS side effects and the therapeutic effects of antipsychotic drugs could not be dissociated since the former effects were assumed to be required for the latter effects [33,59], the recent introduction of more specific second generation antipsychotic drugs implied otherwise [33,60]. Thereby, our findings suggest that whilst the blockade of cardiac HERG1 channels by antipsychotic agents causes acquired LQT side effects, the blockade of neuronal HERG1 channels by these drugs might contribute to their therapeutic effects, as previously proposed by a number of authors [35,61-63]. Consistently, our findings might have considerable impact on antipsychotic treatment in clinical practice. Firstly, *HERG1 *gene variations might predict the efficacy of an antipsychotic drug in a given patient, in addition to determining the predisposition of an individual to acquired LQT syndrome. Secondly, the introduction of more specific antipsychotic drugs that preferentially block neuronal rather than cardiac HERG1 channels might reduce the occurrence of acquired LQT side effects in patients without worsening therapeutic benefits. Thirdly, specific neuronal HERG1 channel modifiers could also be developed for the treatment of SCH in conjunction with antipsychotic drugs in order to improve the therapeutic outcome. Finally, we propose that a specific neuronal HERG modifier, especially for HERG3 or HERG2 channels, might be a more effective and safer pharmacological intervention than others [64] in primary prevention of SCH at high-risk individuals.

## Limitations

One limitation of our study is its moderate sample size. Although the number of our study subjects is generally regarded as small for association studies, we were able to confirm the findings reported in larger populations by Huffaker et al. [35] regarding the association of SCH with *HERG1 *and specifically the *HERG1 *SNP rs3800779. Nevertheless, our study needs to be reproduced in a larger sample size regarding the identification of two previously undescribed haplotypes located on 7q36.1 region encompassing *HERG1 *and more significantly associated with SCH than individual SNP.

A further limitation of our study is the lack of details on the ethnic characteristics of our samples. Indeed, considerable mixtures between ethnicities for many generations prevent to have precise data on the exact ethnic origins of many people in Turkey. Nevertheless, both patients and controls were recruited from Istanbul metropolitan area, and we believe that both populations do well represent the general population of Turkey and that a possibility of stratification between populations does not exist in our samples.

## Conclusions

In summary, we have found that a SNP (rs3800779, c.307+585G>T) located on *HERG1 *shows significant differences in both genotype and allele distributions between patients with SCH and control subjects, and we have also identified two previously undescribed four-marker haplotypes located on 7q36.1 region in chr7:150225599-150302147 bp position encompassing *HERG1 *and more significantly associated with SCH than individual SNP (the at-risk haplotype A-A-A-T and the protective haplotype C-A-C-G, which are nearly allelic opposite of each other). Our results indicate that the potassium channel gene *HERG1 *is associated with SCH, although this needs to be reproduced in a larger sample size and in other ethnic groups. Nevertheless, we believe our findings merit further investigation in order to determine whether the *HERG1 *gene is also a common susceptibility factor to psychosis including affective disorders besides SCH, considering that genetic associations are generally not specific to one of the traditional diagnostic categories of functional psychoses [65]. Finally, as the other two members of the ERG channels family, ERG3 and ERG2, which are known for their widespread expression in brain, are seemingly nervous system-specific [24] and form heterotetramers with ERG1 channels [45-48], we believe that *HERG3 *and *HERG2 *genes also need to be extensively studied in psychotic disorders. Future research, most importantly a combinatoric approach [66] integrating the genetics (genetic variation, genetic regulation and epigenetic modification) and the biology (synthesis, trafficking, gating and conductance) of HERG channels with clinical data, will illuminate their exact *lieu *in the pathobiology and treatment of SCH and psychosis.

## Competing interests

The authors declare that they have no competing interests.

## Authors' contributions

FA involved in the conception of the study, designed and coordinated the study, organized the genetic arm of the study, analyzed the data, interpreted the results and designed and drafted the manuscript. TTA involved in the conception of the study, analyzed the data, interpreted the results and designed and drafted the manuscript. NC participated in the statistical analyses, analyzed the data, interpreted the results and revised the manuscript critically. FO recruited the subjects, made consensus clinical diagnoses, participated in the statistical analyses and revised the manuscript critically. DY recruited the subjects, made consensus clinical diagnoses and revised the manuscript critically. UO interpreted the results and revised the manuscript critically. ST organized the clinical arm of the study, interpreted the results and revised the manuscript critically. All authors read and approved the final manuscript.
